# Programmed cell death in *Saccharomyces cerevisiae* is hampered by the deletion of *GUP1* gene

**DOI:** 10.1186/1471-2180-12-80

**Published:** 2012-05-22

**Authors:** Joana Tulha, Fábio Faria-Oliveira, Cândida Lucas, Célia Ferreira

**Affiliations:** 1 CBMA (Centre of Molecular and Environmental Biology), Department of Biology, University of Minho, Campus de Gualtar, 4710-057, Braga, Portugal

## Abstract

**Background:**

During the past years, yeast has been successfully established as a model to study mechanisms of programmed cell death regulation. *Saccharomyces cerevisiae* commits to cell death showing typical hallmarks of metazoan apoptosis, in response to different stimuli. Gup1p, an *O*-acyltransferase, is required for several cellular processes that are related to apoptosis development, such as rafts integrity and stability, lipid metabolism including GPI anchor correct remodeling, proper mitochondrial and vacuole function, bud site selection and actin dynamics. Therefore, we hypothesize that apoptotic process would be affected by *GUP1* deletion.

**Results:**

In the present work we used two known apoptosis inducing conditions, chronological aging and acetic acid, to assess several apoptotic markers in *gup1∆* mutant strain. We found that this mutant presents a significantly reduced chronological lifespan as compared to Wt and it is also highly sensitive to acetic acid treatment. In addition, it presents extremely high levels of ROS. There were notorious differences on apoptotic markers between Wt and *gup1∆* mutant strains, namely on the maintenance of plasma membrane integrity, on the phosphatidylserine externalization, on the depolarization of mitochondrial membrane and on the chromatin condensation. Those suggested that the mutant, under either condition, probably dies of necrosis and not from apoptosis.

**Conclusions:**

To Gup1p has been assigned an important function on lipid rafts assembly/integrity, lipid metabolism and GPI anchor remodeling. Our results provide, for the first time, the connection of the integrity of yeast lipid rafts and apoptosis induction and/or signaling, giving new insights into the molecular mechanisms underlying this process in yeast.

## Background

Apoptosis is the most common process of programmed cell death (PCD) in eukaryotes. It is vital for the fast elimination of useless or injured cells, and for the differential development of tissues and organs. In humans the malfunction of this process leads to severe diseases, namely neurodegenerative disorders, AIDS and cancer. The existence of PCD processes in lower eukaryotes or bacteria was for long disregarded due to the absence of obvious benefits for unicellular organisms. Nonetheless, numerous works contributed to evidence PCD occurring in single cell organisms [1-4], as well as to the establishment of yeast as a good model to study mechanisms of apoptotic regulation [5,6]. Multicellular aggregates of microbial cells, like colonies or biofilms, are spatially organized and require the specialization of cells differentially localized to ensure supply of nutrients and water to the whole cell ensemble [7]. The growing concept that microbial multicellular aggregates form functional and higher organized structures, as a kind of proto-tissue, supports the notion that PCD may be a much more spread and conserved mechanism of cellular altruistic behaviour.

The characteristic apoptotic markers, as DNA fragmentation, phosphatidylserine externalization, chromatin condensation, release of cytochrome *C*, and/or caspases activation are also valid to assess apoptotic yeast cells [1,8]. Furthermore, an increasing list of homologues of apoptotic regulators in metazoans has been identified in yeast, such as Yca1p, the proposed yeast caspase [9]; Aifp, the apoptosis inducing factor [10]; EndoG, an endonuclease which regulates not only life but also death in yeast [11]; Nma111p, a yeast HtrA-like protein [12]; Bir1p, an inhibitor-of-apoptosis protein [13] and Ybh3p, a yeast protein that interacts with Bcl-xL and harbours a functional BH3 domain [14]. Additionally, the expression in *S. cerevisiae* of the mammalian Bcl-2 family and PKC isoforms [15], led to the same phenotypes observed in mammalian cells, providing evidence that apoptosis is an evolutionarily conserved mechanism. Several agents can induce yeast PCD, like hydrogen peroxide, UV radiation, the absence of nutrients, hyper-osmotic stress, acetic acid [8] and aging [6]. Aging in yeast can be studied assessing either replicative or chronological lifespan. Replicative lifespan is defined as the number of daughter cells a single yeast mother cell produces before senescence; chronological lifespan is defined by the length of time cells can survive in a non-dividing, quiescence-like state [16]. Chronological aged yeast cells also exhibit typical apoptotic markers. During chronological aging, the old yeasts die and release certain substances (nutrients) into the medium in order to promote survival of other aged cells, yet fitter ones [6].

On the other hand, it has been demonstrated that apoptotic *S. cerevisiae* cells display changes in the expression of some genes associated with the sphingolipids metabolism [17], which is consistent with changes in the proportions of the various sphingolipid types in dying cells [18]. Carmona-Guitierrez and co-authors [19] observed the apoptosis induction by external addition of C2-ceramide, whereas Barbosa and co- authors reported changes in sphingolipids during chronological aging, namely a decrease of dihydrosphingosine levels and an increase of dihydro-C(26) -ceramide and phyto-C(26) -ceramide levels [20]. Also, a role in apoptosis and aging of Ydc1p ceramidase was described [18], and a yeast homologue of mammalian neutral sphingomyelinase 2 was associated with apoptosis [21]. Moreover, some intermediates in sphingolipids biosynthesis act as signalling molecules and growth regulators [22,23]. Nevertheless, modest attention has been paid to the involvement of sphingolipids in yeast PCD.

In *S. cerevisiae,* sphingolipids are mainly located in the plasma membrane, being more concentrated along the sphingolipid-sterol rich domains [24], commonly named rafts. These domains play fundamental roles in connecting the plasma membrane to the cytoskeleton, ER and Golgi, and therefore in the correct protein sorting and trafficking through exocytosis/endocytosis [25]. Moreover, rafts harbour signalling molecules besides sphingolipids, like kinases, PI2P (phosphatidylinositol-3,4-diphosphate), and GPI (glycosylphosphatidylinositol)-anchored proteins [25,26]. The latter, are proteins attached to the plasma membrane via a lipid anchor that contains either a ceramide or diacylglycerol [27]. Gup1p is a membrane-bound *O*-acyltransferase [28,29] involved in lipid metabolism, rafts integrity and assembly [30] and GPI anchor remodelling [31]. This protein was primarily identified associated with phenotypes on glycerol metabolism and transport [32], but has further been implicated in a vast number of distinct processes, namely cell wall structure, composition and biogenesis [33], plasma membrane assembly and composition [30,34], cytoskeleton polarization and bud site selection [35], and telomere length [36], all of which directly or indirectly associated with apoptosis. This work presents evidence that cells lacking *GUP1* are not able of undergoing apoptosis, as revealed by the analysis of several apoptotic markers (mainly lack of membrane integrity and of phosphatidylserine externalization). Instead the mutant appears to be experiencing a necrotic cell death process, upon both chronological aging and acetic acid induction. This result adds to the growing view that as in higher eukaryotes, lipids are involved in signalling PCD in yeast.

## Results

*GUP1* is involved in a wide range of cellular processes, some of which are associated directly or indirectly with apoptosis, such as rafts integrity and lipids metabolism [17,18,21,30,31,34], cytoskeleton polarization [35,37], and telomere length [36,38]. In the present work, we assess apoptotic markers for *gup1∆* mutant strain and compare them with Wt, under two different conditions documented to induce apoptosis in yeast: chronological aging and acetic acid [8,39].

### *gup1∆* mutant cells exhibit a reduction in chronological lifespan

Yeast chronological lifespan is described as the length of time a population remains viable in the non-dividing/stationary phase [40,41]. Chronologically aged yeast cells die exhibiting specific markers of apoptosis [6,40]. We checked the survival of *gup1∆* chronologically aged cells in comparison to Wt, continuously for 30 days throughout stationary phase until complete death of the culture.

**Figure 1:**
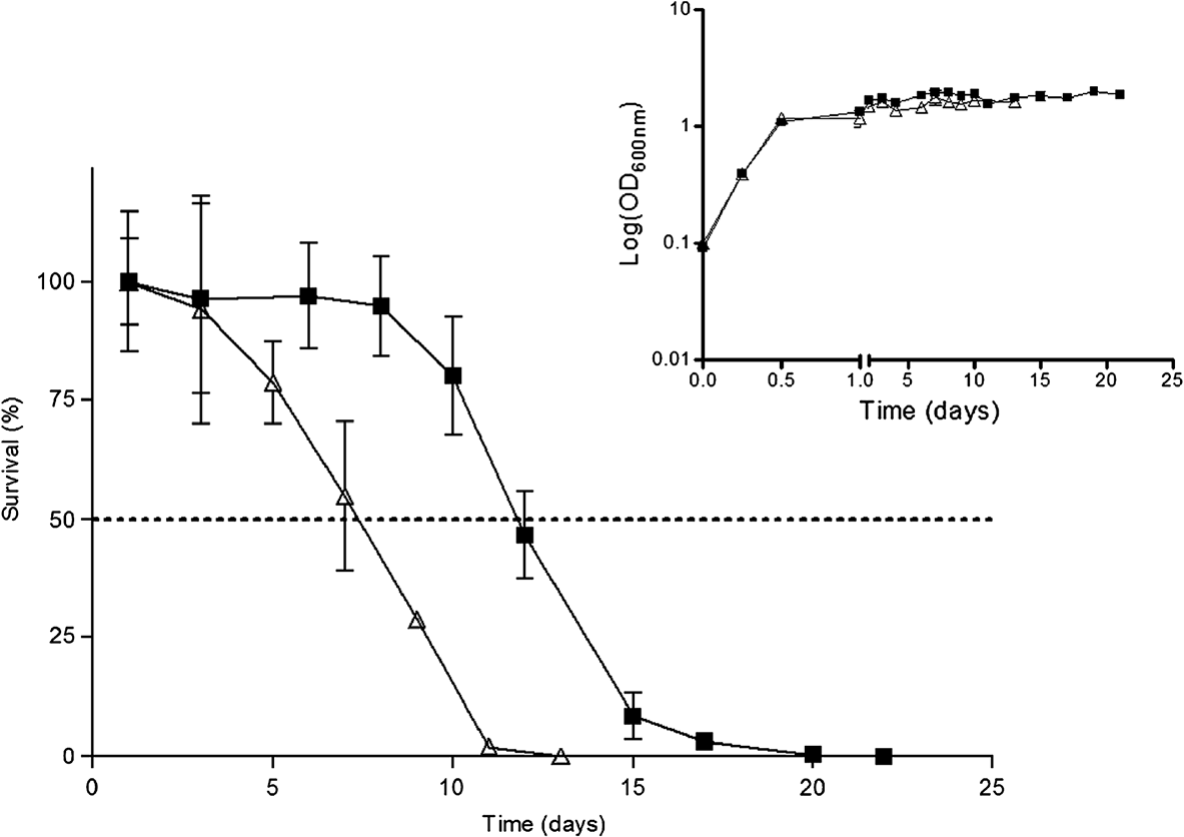
**Deletion of *GUP1* decreases chronological lifespan.** Wt (■) and *gup1∆* mutant (∆).cells were inoculated in YNB medium and survival monitored by c.f.u. for 30 days (100% represents the 1,000 plated cells counting using a Neubauer chamber). The growth curve in YNB for both strains is presented in the insert. Data represent mean ± SD of at least 3 independent experiments.

The growth curve (Figure 1 insert) showed an apparent similar growth rate for both strains during exponential phase, as well as an almost coincident transition to diauxic and stationary phases. On the other hand, the survival curve (Figure 1) showed that *gup1∆* mutant cells died considerably sooner than Wt. After day 3 the survival rate of *gup1∆* mutant started to decrease, reaching 50% around day 7, and in day 11 we observed that only a small number of *gup1∆* mutant cells stayed alive. Conversely, Wt strain begins to die around day 8, reaches 50% survival at day 12 and on day 19 the culture was practically dead.

### Chronological aged *gup1*∆ mutant seems to be incapable of dying by apoptosis but rather by necrosis

In order to investigate whether chronologically aged Wt and *gup1∆* mutant cells die by apoptosis, we analyzed several apoptotic markers in exponentially growing and chronologically aged cultures on both strains [6,42]. We choose 6 hours growth to assess exponential cells, and day 7 or day 12 to test chronologically aged cells of *gup1∆* mutant and Wt, respectively.

**Figure 2:**
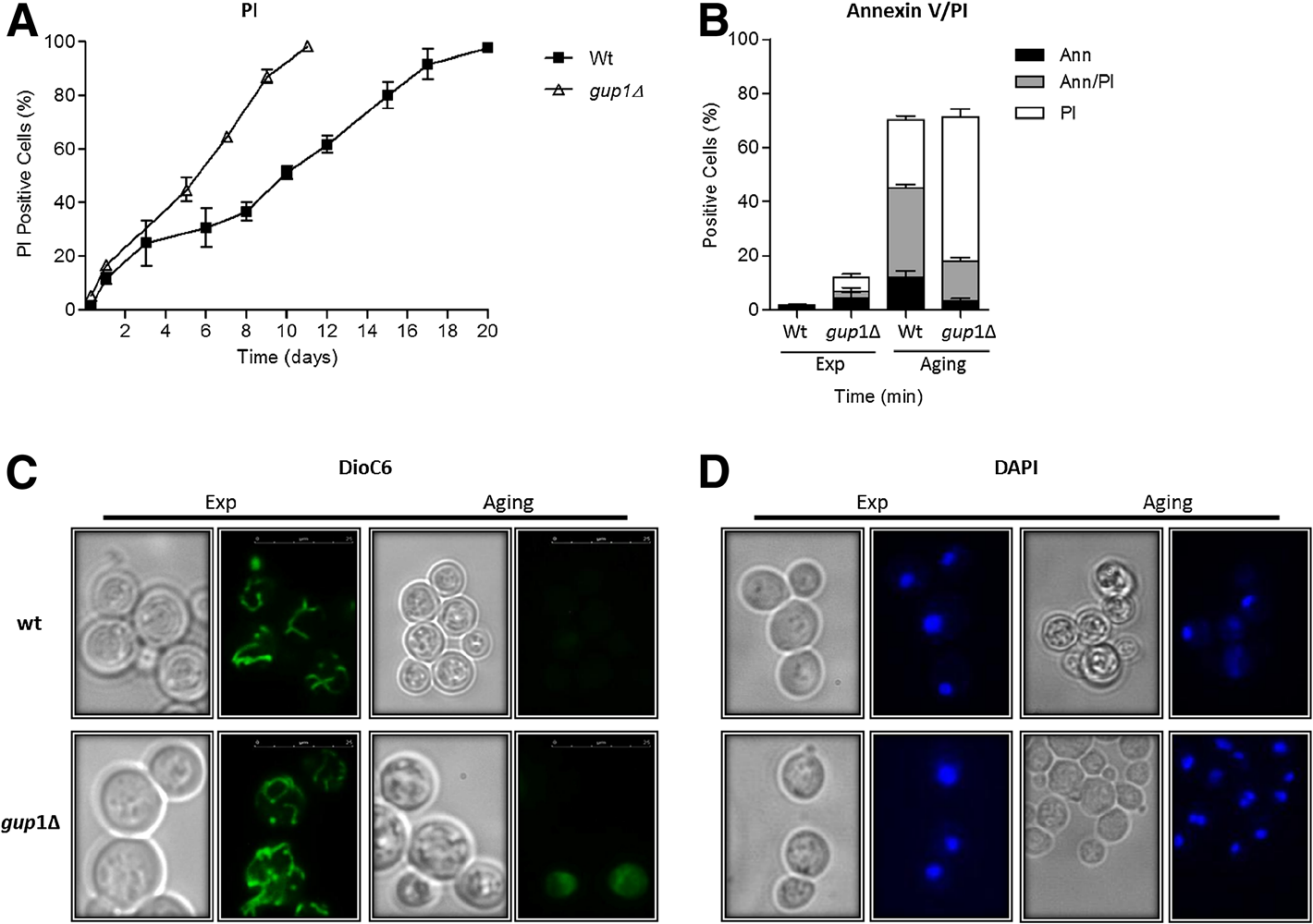
**Analysis of apoptotic markers in Wt and *gup1* .***∆***chronologically aged cells.** (**A**) Graphic representation of the percentage of cells displaying positive PI staining. (**B**) Phosphatidylserine externalization assessed by cytometric analysis of Annexin V labelling. Graphic representation of the percentage of cells displaying Ann V (+)/PI (−) (black bars), Ann V(+)/PI (+) (grey bars) and Ann V(−)/PI (+) (white bars). (**C**) Representative photos of DiOC_6_ staining exponential phase and aged cells. (**D**) Representative photos of DAPI staining of exponential phase and aged cells. For flow cytometry and fluorescence microscopy assays a minimum of 35,000 and 300 cells were counted, respectively. Data represent mean ± SD of 3 independent experiments.

In yeast, as in mammalian cells, the maintenance of plasma membrane integrity during cell death is an indicator of apoptosis. In this work, we evaluated the integrity of plasma membrane, in exponential and aged Wt and *gup1∆* mutant strains, by PI staining. In *gup1∆* mutant, we observed a substantial increase in the number of cells stained with PI over time, until every cell presented PI positive. Still, although the pattern is identical, in Wt the percentage of PI positive cell was proximally 2-fold less (Figure 2A). Yet, the percentages of PI positive cells can be over evaluated since apoptotic cells can become leaky during further cultivation, increasing PI positives. To distinguish this secondary necrosis from primary necrosis further tests were performed.

Phosphatidylserine has an asymmetric distribution in the lipid bilayer of the cytoplasmic membrane [43]. The exposure of phosphatidylserine at the outer surface of the cytoplasmic membrane occurs at the early stages of apoptosis [44], when membrane integrity is still retained. We checked this through the FITC-coupled Annexin V reaction followed by flow cytometry of co-labeled Annexin V/PI cells. We observed that *gup1∆* mutant aging cells presents a significant percentage (53%) of necrotic cells (Ann (−)/PI(+)). In contrast, in Wt cells the exposure of phosphatidylserine (Ann (+)/PI (−)) increased in aged cells (less than 1% to ~12%) (Figure 2B).

In order to evaluate the mitochondrial membrane depolarization, DiOC_6_ was used. At a concentration of 20 ng/ml this dye accumulates specifically at mitochondrial membranes and can be observed by fluorescence microscopy. Nonetheless, cells that have low mitochondrial membrane potential will fail to accumulate DiOC_6_[37]. Both *gup1∆* mutant and Wt exponential cells stained with DiOC_6_ revealed intact mitochondrial networks, confirming a normal polarization of mitochondrial membranes (Figure 2C left panels). Aged cells (7 and 12 days in *gup1∆* mutant and Wt, respectively), showed a decrease in green fluorescence of approximately 40% in Wt and 50% in *gup1∆* mutant, reflecting a reduction in mitochondrial membrane potential (Figure 2C right panels). Moreover, some cells exhibited a strong green fluorescence all over the cell, mainly in *gup1∆* mutant strain, suggesting that these cells possibly had the plasma membrane altered, which in turn resulted in the accumulation of DiOC_6_ on the cytosol (Figure 2C right panels).

Finally, we evaluated chromatin condensation through DAPI staining (Figure 2D). Moderate chromatin condensation upon DAPI staining was observed in 80% of old *gup1∆* mutant cells, which can be visualized by the fluorescent semicircles formed by the chromatin fragments (Figure 2D right panels). Regarding Wt aged cells, we observed some cells with chromatin condensation, but we also detected cells without stained nucleus or even with multiples nucleus (Figure 2D right panels). These are probably due to an endomitosis process [45,46]. In contrast, in exponentially growing cultures, both Wt and *gup1∆* mutant cells presented integral chromatin mirrored as single round fluorescent circles in the middle of the cell (Figure 2D left panels).

### *gup1∆* mutant cells are sensitive to acetic acid

**Figure 3:**
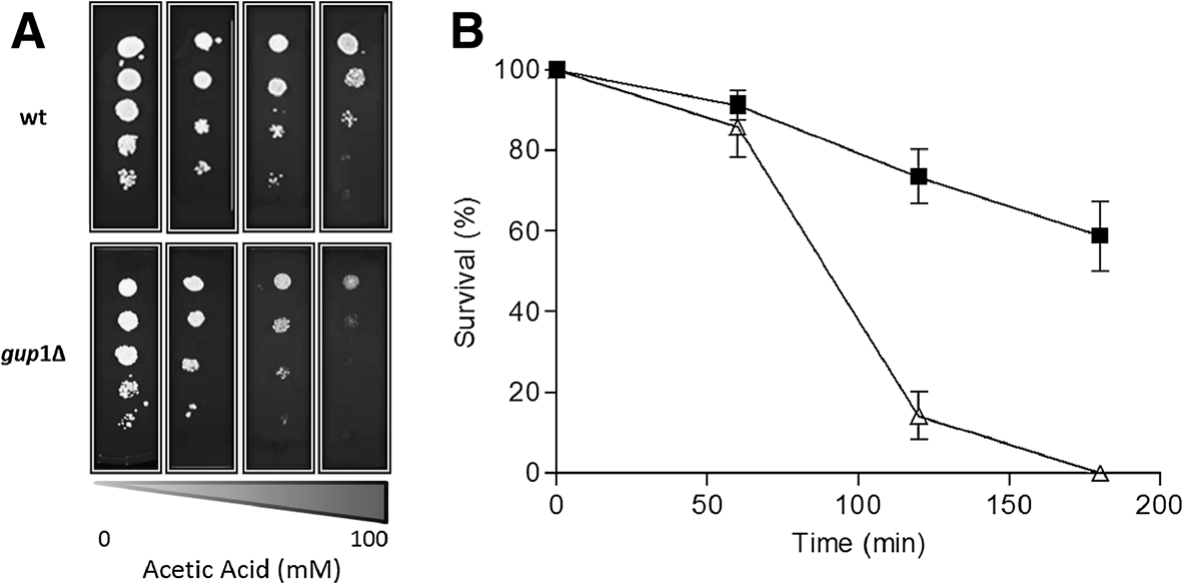
**Loss of *GUP1* confers sensitivity and reduces survival in presence of acetic acid.** (**A**) Sensitivity of Wt and *gup1∆* mutant cells to several increasing concentrations of acetic acid by Dropout assay. Cultures were grown to mid-exponential phase in YNB medium, and ten-fold serial dilutions were spotted onto YNB plates supplemented with acetic acid. All plates were incubated at 30°C for 48 h. (**B**) Survival curve of Wt (■) and *gup1∆* (∆) cultures during acetic acid treatment. Exponential cells were treated with 160 mM acetic acid for 180 min and viability determined by c.f.u. at the indicated time points (100% survival corresponds to the total c.f.u. at time zero). Data represent mean ± SD of at least 3 independent experiments.

In a previous work, it was described that *gup1∆* mutant cells were sensitive to weak acids [33]. However, the concentrations of acetic acid that induce apoptosis in yeast are considerably higher than the ones studied at that time (50 mM). Therefore, we investigated *gup1∆* mutant and Wt sensitivity to a wide range of acid concentrations (50, 80 and 100 mM). With the lowest concentration of acetic acid (50 mM), no effect was observed; however, when the concentration was increased both strains were affected, being *gup1∆* mutant strain the most sensitive one. At the highest concentration tested, 100 mM of acetic acid, the difference between the two strains was more obvious, with *gup1∆* mutant showing growth only until the second dilution, whereas Wt presents growth up to the fourth dilution (Figure 3A). Additionally, we determined the death kinetics of both strains treated with 160 mM of acetic acid (Figure 3B), as commonly assayed to evaluate apoptosis induced by acetic acid [4]. For that, Wt and *gup1∆* mutant cells at exponential growth phase were exposed to acetic acid, and the survival rate measured by c.f.u. counts. In both cases, the yeast cells died in response to acetic acid, but the cell death patterns were different. Until 60 min of acetic acid treatment, no significant difference was found between Wt and *gup1∆* mutant strains, presenting around 90% and 85% cell viability, respectively. These percentages progressively decreased in both strains, being this reduction more accentuated in the *gup1∆* mutant strain. After 120 min in the presence of acetic acid, only 15% of *gup1∆* mutant cells remained alive, whereas Wt presented a survival rate of around 75%. At the last time-point analyzed, 180 min, the dissimilarity among strains sharpened up; only a few cells of *gup1∆* mutant strain were viable, whereas Wt strain displayed a survival rate of around 55% (Figure 3B).

### Acetic acid induces cell death by necrosis similar to that triggered by chronological aging in the *gup1∆* mutant strain

**Figure 4:**
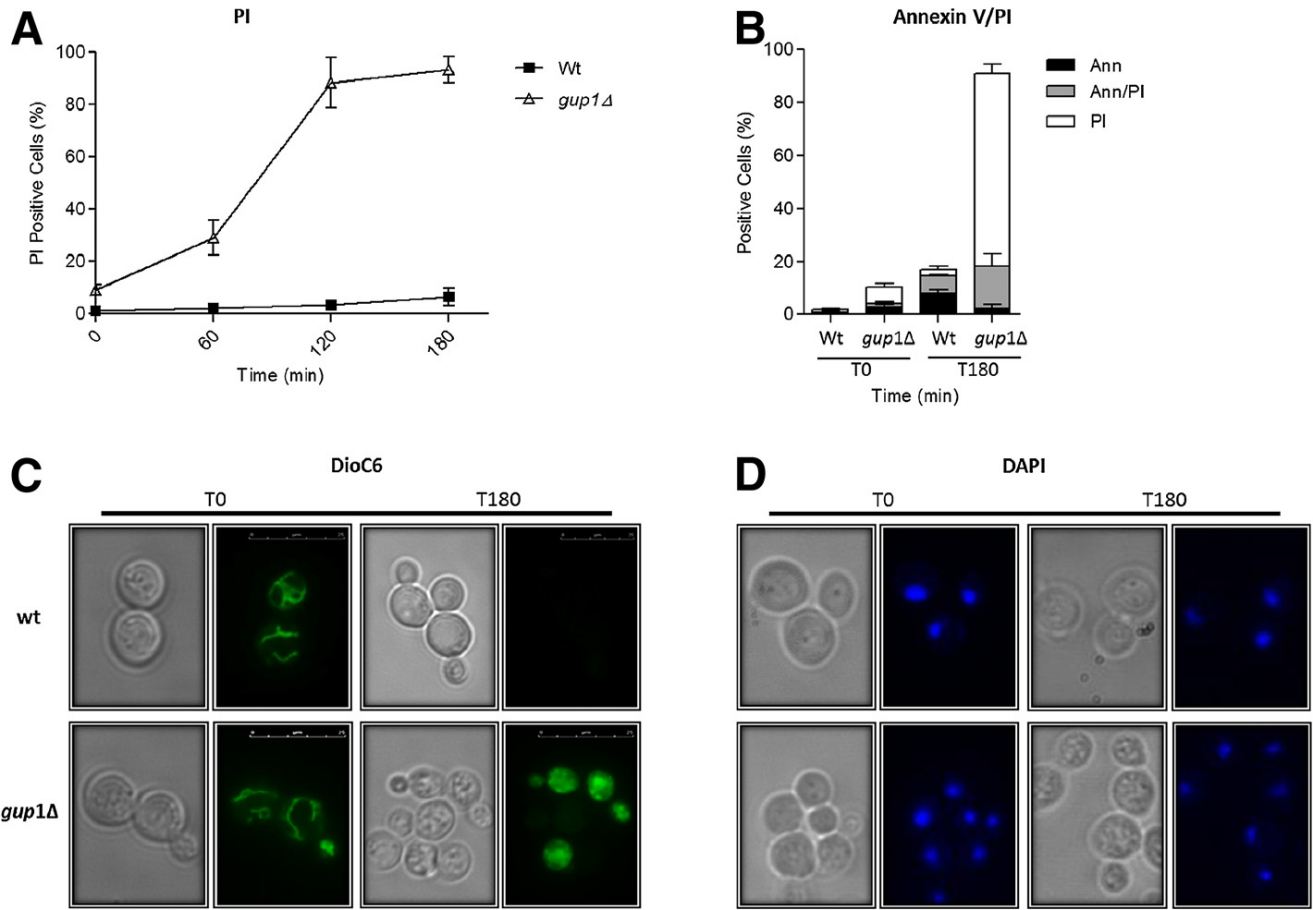
**Analysis of apoptotic markers in cells treated with acetic acid.** Wt and *gup*1∆ mutant cells were exposed to 160 mM acetic acid for 180 min. (**A**) Graphic representation of the percentage of cells displaying positive PI staining. (**B**) Phosphatidylserine externalization assessed by cytometric analysis of Annexin V labelling. Graphic representation of the percentage of cells displaying Ann V (+)/PI (−) (black bars), Ann V(+)/PI (+) (grey bars) and Ann V(−)/PI (+) (white bars). (**C**) Representative photos of DiOC_6_ staining untreated cells and cells after 180 min at acetic acid treatment. (**D**) Representative photos of DAPI staining untreated cells and after 180 min acetic acid treatment. For flow cytometry and fluorescence microscopy assays a minimum of 35,000 and 300 cells were counted, respectively. Data represent mean ± SD of 3 independent experiments.

In order to assess whether cell death induced by acetic acid treatment followed a programmed process of apoptosis, we analyzed several apoptotic markers. The first marker analyzed was PI staining to estimate the loss of membrane integrity. Acetic acid treatment led to a pronounced increase of *gup1∆* mutant PI positive cells, reached nearly 100% after 180 min of treatment, while in the Wt strain this percentage did not exceed 10% (Figure 4A). In addition, we examined the phosphatidylserine exposure by simultaneously FITC- coupled Annexin V/PI staining (Figure 4B). Similarly to what was observed with the aging experiment, a substantial increase (72%) in necrotic cells (Ann (−)/PI(+)) were observed after treatment with acetic acid (180 min treatment). In opposition, Wt strain presents an increase (8%) in apoptotic cells (Ann (+)/PI(−)) after the treatment with acetic acid (Figure 4B).

Yeast mitochondria undergo both structural and functional changes after the incubation with acetic acid [47], including mitochondrial membrane depolarization. In order to evaluate this phenomenon, DiOC_6_ staining was used to visualize mitochondrial membranes (Figure 4C). Just before apoptosis induction with acetic acid, most of Wt and *gup1∆* mutant cells presented intact mitochondrial networks (Figure 4C left panels). After the treatment, it was possible to visualize depolarization of mitochondrial membranes in approximately 40% and 30% of *gup1∆* mutant and Wt cells, respectively, mirrored by the absence of fluorescence (Figure 4C right panels). Furthermore, we observed a considerable number of *gup1∆* mutant cells displayed an increase in DiOC_6_ green fluorescence, similarly to the results obtained when the apoptotic inductor was chronological aging (Figure 4C right panels).

Additionally, we checked for chromatin condensation during acetic acid treatment by staining cells with DAPI (Figure 4D). Nearly no chromatic condensation was observed in both *gup1∆* mutant and Wt untreated cells, as reflected by the single round fluorescent circles in the center of the cells (Figure 4D left panels). Yet, after the treatment with acetic acid, we observed a significant increase in *gup1∆* mutant cells exhibiting moderate chromatin condensation along the nuclear envelope (~90%). In Wt, ~25% of cells presented chromatin condensation (Figure 4D right panels).

### *gup1∆* mutant cells accumulate large amounts of ROS during chronological aging and acetic acid treatment

**Figure 5:**
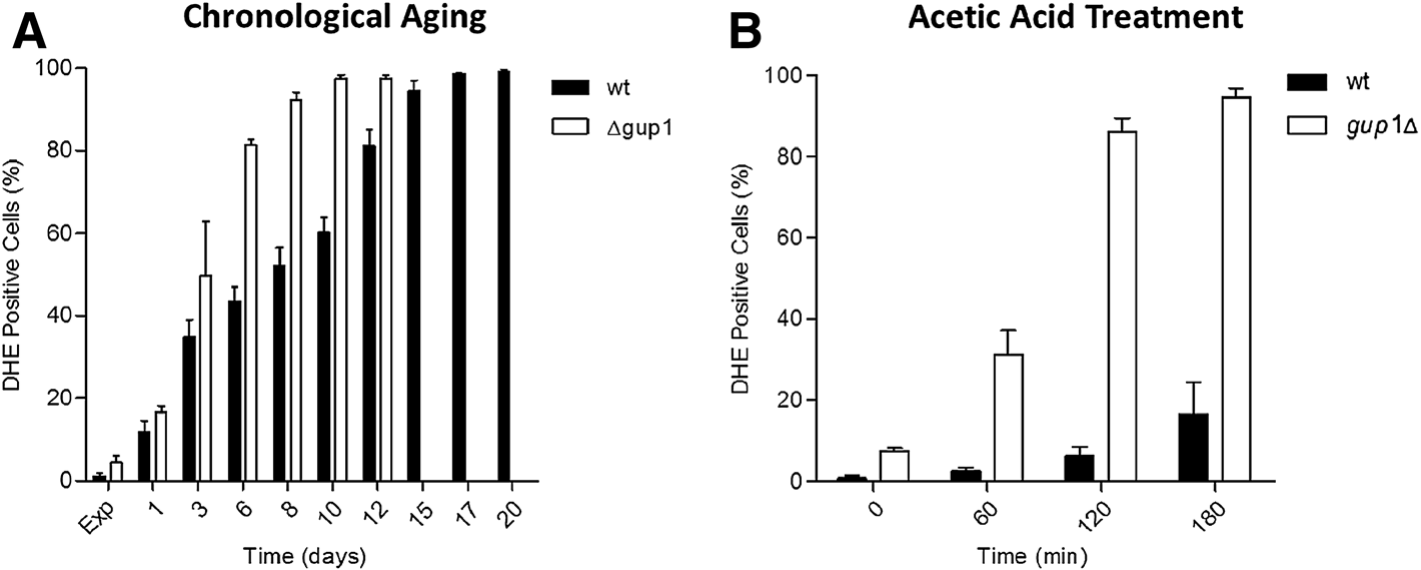
** *GUP1* deletion promotes substantial ROS accumulation.** Cells from chronological lifespan assay (**A**) and from acetic acid treatment (**B**) were analyzed for accumulation of ROS using DHE staining by flow cytometry. At least 35,000 cells were analyzed. Data represent mean ± SD of at least 3 independent experiments.

It is well documented that the loss of mitochondrial membrane potential can lead to increased production of ROS in higher eukaryotes, which is seen as an apoptotic-related process in yeasts [3,46]. On the other hand, several points of evidence indicate that, in yeast, the accumulation of ROS is a major factor determining aging [48,49] and triggering PCD [3,39,50]. The accumulation of ROS is commonly measured by incubating cells with dihydroethidium (DHE), which is oxidized (by ROS) to the ethidium. ROS were measured on both chronologically aged and acid acetic treated *gup1∆* mutant and Wt cells. Regarding chronological assay, this covered exponential, stationary and death phases (Figure 5A). A significantly higher increase of ROS levels over time was observed in *gup1∆* mutant in comparison to Wt cells. The biggest difference was on day 6 (stationary phase), when the percentage of *gup1∆* mutant cells exhibiting ROS accumulation was the twice (~80%) that of Wt cells (~40%). The mutant reached 100% of cells with ROS accumulation on day 10, while Wt took 17 days to reach that state (Figure 5A). Still regarding *gup1∆* mutant, the 100% ROS was maintained till the end of experiment (more five days), which is in agreement with the observed death of these strain cells (Figure 1 - after 12 days more than 99% death). The difference between Wt and *gup1∆* mutant strains was also extremely notorious in acetic acid treated cells (Figure 5B). Soon after acetic acid addition, *gup1∆* mutant exhibited ROS accumulation in ~ 8% of the cells, whereas Wt presented less than 1%. This difference was accentuated with time. At one hour treatment *gup1∆* mutant cells with ROS accumulation was higher than 30% and Wt cells less than 5%. Two hours treatment led to a substantial rise of ROS positive *gup1∆* mutant cells (~85%) compared with only ~10% of Wt. At the end of the treatment, almost all *gup1∆* mutant cells exhibited ROS accumulation, in clear contrast with the ~15% of ROS accumulation displayed by Wt strain (Figure 5B).

## Discussion

The finding of an endogenous PCD process with an apoptotic phenotype has turned yeast into a powerful model for apoptosis research [39,51,52]. In fact, *S. cerevisiae* commits to cell death showing typical features of mammalian apoptosis, in response to different stimuli. However, how cell compounds participate in the processes leading to cell death in yeast remains to be established. Gup1p, an *O*-acyltransferase, is required for several cellular processes that are related to apoptosis development, namely, rafts integrity and stability, lipid metabolism including GPI anchor correct remodeling, proper mitochondrial and vacuole function, and actin dynamics [30,31,33,35,37,42,53-56].

In this work we used two known apoptosis-inducing conditions, chronological aging [6] and acetic acid [4], to assess several apoptotic markers in *gup1∆* mutant strain. We found that, when compared with Wt, *gup1∆* mutant presents a significant reduced chronological lifespan, showing almost no viability after 11 days incubation. Chronologically aged yeast cultures were shown to die exhibiting typical apoptotic markers [6]. Accordingly, we showed that chronologically aged Wt cells predominantly commit to an apoptotic death, as revealed by a) PI negative cells; b) phosphatidylserine externalization; c) depolarization of mitochondrial membrane; and d) chromatin condensation. Yet, *gup1∆* mutant aged cells seem to be incapable of undergoing apoptosis. Instead, these cells appeared to be experiencing a necrotic cell death process. The *gup1∆* mutant aged culture exhibited a higher number of cells with loss of membrane integrity, and did not reveal an increase of phosphatidylserine exposure on the surface of the plasma membrane. Such observations discredit the possibility that these cells are dying through an apoptotic process, being more likely that the reduction in lifespan is due to a necrotic death. Furthermore, both loss of mitochondrial membrane potential and moderate chromatin condensation that we observed in this mutant have already been described in necrotic phenotypes [57,58]. Lately, several points of evidence suggest that necrotic cell death also occurs in yeast. Moreover, that can occur under normal physiological conditions or in the presence of cell death inducing substances, and not necessarily resulting from brutal chemical or physical damage, as previously thought [11].

We also used acetic acid as an apoptotic inducer of cell death in both Wt and *gup1∆* mutant strains. Our results revealed that acetic acid induces a cell death process similar to that observed in aging cultures. These results are in accordance with the hypothesis proposed in a previous work, in which the toxicity of acetic acid produced during aging was suggested as the major cause of chronological aging in yeast [59]. Reinforcing such idea are the acidified cultures that we observed during aging, probably resulting from acetic acid production and release to the medium (data not shown). Moreover, it was also reported that the signaling of acetic acid-induced apoptosis is linked to amino-acid metabolism as well as to the TOR pathway [60], as it happens in the aging process [61]. A necrotic death induced by acetic acid was already observed in other yeast mutants, namely in mutants in class C VPS genes that code for proteins essential for vacuolar and endossomal vesicle function [42].

Accumulation of ROS has predominantly been associated to yeast apoptosis under numerous conditions [62-64]. Some studies have addressed a fundamental role of ROS on the execution apoptotic death, after treatment with low doses of hydrogen peroxide [3] and on the superoxide-mediated altruistic program of aging [65]. Interestingly, however, many studies have suggested a crucial involvement of ROS during necrosis of mammalian cells [66] as well as in yeast necrosis [42,64]. This evidence is in accordance with our results. We observed a significant difference in ROS accumulation between Wt and *gup1∆* mutant strain in both chronological aging and acetic acid treatment. *gup1∆* mutant cells, which present a necrotic phenotype, have an extremely higher accumulation of ROS. If ROS can contribute, apart from its role on apoptosis, to the necrotic cell death in yeast as well, or if it is rather a byproduct that accumulates as a result of cellular demise, remains to be elucidated.

Gup1p has been described to have an important function on lipid rafts assembly/integrity [30]. In the literature, rafts have been increasingly implicated on regulation of apoptotic signaling in mammalian cells [54,67]. In response to intra or extracellular stimuli, lipid rafts can include or exclude proteins to variable extents. This favors specific protein-protein interactions and modulates the activity of signalling apoptotic cascades. Moreover, in mammalian cells a number of proteins involved in apoptotic signals have been found to locate in lipid rafts, namely Fas/CD95 receptor [68] and the pro-apoptotic protein of Bcl-2 family, Bad [69]. Our results showed that the PCD processes in *S. cerevisiae* is altered by *GUP1* deletion and reinforce the importance of lipid rafts on the regulation of apoptotic signaling in yeast. Moreover, our findings point to that these membrane domains seem to be indispensable for a proper development of PCD, under aging and acetic acid conditions, namely in the switch from a necrotic to an apoptotic death phenotype.

## Conclusions

We demonstrate that *gup1∆* mutant strain present a significantly reduced chronological lifespan comparing to Wt. Moreover, this mutant showed to be highly sensitive to acetic acid. Yet, while chronologically aged and acetic acid treated Wt cells die exhibiting apoptotic markers, *gup1∆* mutant cells under the same conditions seems to be incapable of undergoing apoptosis. Instead, these cells appeared to be experiencing a necrotic cell death process. In addition, those cells also present extremely high levels of ROS. Being *gup1∆* mutant affected in lipid rafts integrity/assembly, lipid metabolism and GPI anchor remodeling we propose that the integrity of rafts may be essential for apoptosis induction and/or signaling. This provides for the first time the possible participation of lipid rafts in yeast apoptosis, giving new insights into the molecular mechanisms underlying this particular process of PCD, and highlighting the complex network of cellular structures that interact, cooperate and compete to regulate cell death.

## Methods

### Strains and growth conditions

The *Saccharomyces cerevisiae* strains used in this study were W303-1A [70] and BHY54 [32]. Yeast batch cultures were grown aerobically in minimal medium (0.67% (wt/v) YNB (Difco)) with 2% (wt/v) glucose and adequate quantities of auxotrophic requirements [71]. Incubation was performed at 30°C, 200 rpm, orbital shaking and air/liquid ratio 3/1. Yeast strains maintenance was done on rich medium (YPD (Difco) with 2% agar), grown at 30°C for 48 h and kept at 4°C up to 5 days.

### Chronological lifespan

For chronological lifespan experiments, pre-inoculum cultures grown overnight on YNB were used to start batch cultures at 0.05 (OD_600nm_) in fresh YNB medium. At the stipulated time points, culture aliquots were taken to assess growth through OD_600_ and colony forming units (c.f.u.), and for apoptotic assays. c.f.u. were determined plating 1,000 cells, counted on a Neubauer chamber, on YPD agar, as previously described [6]. Colonies were counted after 48 h incubation at 30°C. No further colonies appeared after that incubation period.

### Sensitivity to acetic acid

Dropout tests were performed from mid-exponential YNB cultures containing approximately 1 × 10^6^ cells/ml. Ten-fold serial dilutions were made, and 5 μl of each suspension was applied on YNB medium supplemented with different acetic acid concentrations (50, 80 and 100 mM). Results were scored after 48 h incubation at 30°C.

### Acetic acid treatment

Yeast strains were grown until exponential phase (OD_600_ = 0.5–0.6) on YNB medium. Then the cultures were collected and resuspended to a final concentration of 10^7^ cells ml^-1^ in fresh YNB adjusted to pH 3.0 with HCl and containing 160 mM acetic acid. Incubation took place for 180 min at 30°C as previously described [4,72]. At determined time points, 40 μl from a 10^−4^ cell suspension were inoculated onto YPD agar plates and c.f.u. were counted after 48 h incubation at 30°C. The percentage of viable cells was estimated considering 100% survival the number of c.f.u. obtained in time 0.

### Apoptotic markers

PI, Annexin V, DAPI and DiOC_6_ staining were performed both in cells treated with acetic acid and in aging cells as previously described, with some modifications [1,3,4,37].

Membrane integrity was assessed by PI (Propidium Iodide) staining. Cells were harvested, washed and resuspended in PBS (137 mM NaCl; 2.7 mM KCl; 100 mM Na_2_HPO_4_; 2 mM KH_2_PO_4_; pH 7.4) containing PI (4 μg/ml) (Sigma). The samples were incubated for 10 min at room temperature in the dark and analyzed in an Epics® XL™ (Beckman Coulter) flow cytometer. At least 20,000 cells from each sample were analyzed.

Phosphatidylserine exposure was detected by an FITC-coupled Annexin V reaction with the ApoAlertAnnexin V Apoptosis Kit (CLONTECH Laboratories). For that, cells were primarily harvested and washed in digesting buffer (1.2 M sorbitol; 0.5 mM MgCl_2_; 35 mM K_2_HPO_4_; pH 6.8). To promote the drug course through cell wall, an incubation step with Zymolyase (20 T) at 30°C was performed. Phase-contrast microscopy was used to monitor that step, preventing this way damage to the unfixed spheroplasts. Cells were subsequently centrifuged (10 min at 1500 rpm) and resuspended in 200 μl of binding buffer (1.2 M sorbitol; 10 mM HEPES/NaOH, pH 7.4; 140 mM NaCl; 2.5 mM Cacl_2_). To 40 μl of this cell suspension, 2 μl Annexin V (1 μg/ml) and 1 μl PI (4 μg/ml) were added, and the mixture incubated for 20 min at room temperature in the dark. Finally, extra 400 μl of binding buffer were added to the mixture just prior to analysis in an Epics® XL™ (Beckman Coulter) flow cytometer. At least 20,000 cells from each sample were analyzed.

For evaluation of mitochondrial potential the probe DiOC_6_ (3,3′dihexyloxacarbocyanine iodide) (Invitrogen) was used. Cells were harvested, washed, and resuspended in DiOC_6_ buffer (10 mM MES; 0.1 mM MgCl_2_; 2% (wt/v) glucose, adjusted to pH 6 set with Ca(OH)_2_) containing DiOC_6_ (20 ng/ml). Cells were visualized by light microscopy (LM) after 30 min at room temperature in the dark. At least, 300 cells selected randomly were counted per sample. The number of cells counted with mitochondrial depolarization (cells without fluorescence) was indexed to our 100% (300 cells).

Chromatin condensation was assessed by DAPI (4,6-diamino-2-phenylindole dihydrochloride) (Sigma) staining. Cells were harvested, washed, fixed for 45 min with 3.7% formaldehyde, permeabilized with a solution of 70% (v/v) ethanol for 30 min, sonicated for 5 sec and afterwards stained with DAPI (1 μg/ml). Cells were visualized by LM after 5 min at room temperature in the dark. At least 300 cells selected randomly were counted per sample. The number of cells counted with chromatin condensation was indexed to our 100% (300 cells).

Stained cells were visualized in a Leica Microsystems DM-5000B epifluorescence microscope with appropriate filter settings using a 100× oil-immersion objective. Images were acquired with a Leica DCF350FX digital camera and processed with LAS AF Leica Microsystems software.

### Assessment of ROS

To visualize accumulation of ROS cells were harvested by centrifugation, resuspended in PBS in the presence of DHE (Dihydroethidium) (4 μg/ml), and further incubated in the dark for 30 min at room temperature. To quantify the number of cells displaying high ROS levels, at least 20,000 cells were counted in an Epics® XL™ (Beckman Coulter) flow cytometer.

## Authors’ contributions

JT and FF-O carried out the experimental studies, having contributed 75% and 25% respectively. CF supervised JT and FF-O and checked the data. JT and CF wrote this manuscript. CL revised the manuscript. All authors read and approved the final manuscript.
